# Anthropometric, Functional, and Haemodynamic Changes in Bariatric Surgery Patients Within the Polish KOS-BAR Pathway: A Retrospective Cohort Study

**DOI:** 10.3390/diagnostics16111736

**Published:** 2026-06-04

**Authors:** Michalina Damrath, Martyna Hromiak, Bartosz Wilczyński, Katarzyna Gierat-Haponiuk

**Affiliations:** 1 Department of Clinical Physiotherapy, Medical University of Gdańsk, 80-210 Gdańsk, Poland; michalina.damrath@gumed.edu.pl (M.D.); hromiak.m@gumed.edu.pl (M.H.); katarzyna.gierat-haponiuk@gumed.edu.pl (K.G.-H.); 2 Department of Immunobiology and Environmental Microbiology, Medical University of Gdańsk, 80-210 Gdańsk, Poland

**Keywords:** obesity, bariatric surgery, physiotherapy, prehabilitation, rehabilitation, KOS-BAR, 6 min walk test

## Abstract

**Background/Objectives:** Bariatric surgery is an effective treatment for severe obesity. Although physiotherapy delivered before and after surgery may support functional recovery, evidence describing real-world multidisciplinary bariatric pathways with embedded perioperative physiotherapy remains limited. This study evaluated perioperative changes in anthropometric, functional, and haemodynamic outcomes in adults who completed preoperative and postoperative physiotherapy within the multidisciplinary Polish KOS-BAR bariatric pathway. **Methods:** We conducted a single-centre retrospective medical-record study at the University Clinical Centre in Gdańsk. The analysis included a complete-case cohort of 91 adults who completed both supervised physiotherapy cycles and had paired outcome data available. Assessments were performed at four time points: before and after prehabilitation (T1–T2) and before and after postoperative rehabilitation (T3–T4). Outcomes included body mass, body mass index (BMI), waist and chest circumference, 6 min walk test (6MWT) distance with Borg-rated exertion, and haemodynamic measures (heart rate, blood pressure, oxygen saturation) recorded before and after the 6MWT. **Results:** From T1 to T4, body mass decreased by a median of 26.0 kg and BMI by 8.98 kg/m^2^, with reductions in waist (−19.0 cm) and chest (−13.0 cm) circumference. Exercise tolerance improved (6MWT median change +30.0 m), and post-test perceived exertion decreased (median −1.0 point). Pre-6MWT resting HR, post-6MWT HR, and blood pressure decreased. The 6MWT distance increased after prehabilitation (T1 → T2) and again after postoperative rehabilitation (T3 → T4). Exploratory correlations suggested weak nominal associations between greater weight loss and larger 6MWT improvement (Spearman r = 0.251, *p* = 0.016) and between BMI reduction and 6MWT improvement (r = 0.226, *p* = 0.031), but these associations did not remain statistically significant after Bonferroni correction. **Conclusions:** In this retrospective cohort of programme completers, participation in the multidisciplinary KOS-BAR pathway with embedded preoperative and postoperative physiotherapy was associated with improved anthropometric, functional, perceived-exertion, and haemodynamic outcomes across follow-up. Because of the uncontrolled retrospective design, these findings cannot establish the independent effect of physiotherapy relative to surgery and other components of multidisciplinary care. Prospective controlled studies are needed to clarify causality and long-term durability.

## 1. Introduction

Obesity is a chronic metabolic disease and a major public health challenge. According to the World Health Organization, in 2022, 43% of adults worldwide were overweight and 16% had obesity, highlighting the growing clinical and healthcare burden associated with excessive body weight [1,2,3]. Obesity is associated with multiple comorbidities, particularly type 2 diabetes [4,5,6], cardiovascular disease [7,8,9], obstructive sleep apnea [10,11,12,13], and musculoskeletal disorders, and it contributes to reduced mobility, lower exercise tolerance, and impaired daily functioning [14,15,16].

For patients with severe obesity, bariatric surgery remains one of the most effective treatment options, producing substantial weight loss and improving obesity-related comorbidities, quality of life, and long-term health outcomes [17,18]. However, bariatric care does not end with surgery alone. Contemporary treatment pathways are multidisciplinary and may include dietary management, psychological support, medical optimisation, and structured physiotherapy before and after surgery [19,20].

With the growing use of bariatric surgery, increasing attention has been directed toward perioperative strategies that may improve functional reserve and support recovery [21,22,23]. Prehabilitation is intended to enhance physiological capacity before surgery and may improve postoperative outcomes, including functional status and quality of life [24,25,26]. Postoperative rehabilitation aims to restore or improve physical function, support recovery, and facilitate return to daily activity through structured exercise, mobilisation, education, and monitoring [23,27].

In bariatric populations, structured exercise and physiotherapy may plausibly improve walking capacity and perceived exertion by enhancing functional performance and tolerance to activity, while favourable changes in body mass and cardiovascular load across the perioperative period may also be reflected in heart rate and blood pressure outcomes [28,29]. However, most previous studies have evaluated prehabilitation and postoperative rehabilitation as separate interventions. Evidence describing a continuous perioperative physiotherapy pathway delivered both before and after bariatric surgery under real-world clinical conditions remains limited [21,24,30,31,32,33].

Although bariatric surgery is well established as an effective treatment for severe obesity, less is known about real-world functional, perceived-exertion, and haemodynamic trajectories in patients completing structured preoperative and postoperative physiotherapy within a multidisciplinary bariatric care pathway. In the Polish KOS-BAR programme, physiotherapy is embedded within broader surgical, medical, dietary, psychological, and educational care. Describing routinely collected outcomes from this pathway may help identify clinically relevant changes, inform optimisation of perioperative rehabilitation standards, and guide the design of future prospective controlled studies.

Therefore, the aim of this retrospective cohort study was to evaluate changes in functional capacity, haemodynamic responses, and anthropometric outcomes in patients undergoing bariatric surgery who completed preoperative and postoperative physiotherapy embedded within the multidisciplinary Comprehensive Specialist Care in Bariatric Treatment programme (KOS-BAR).

We hypothesised that, among programme completers, participation in this multidisciplinary perioperative pathway would be associated with improved exercise tolerance, reflected by greater 6 min walk test distance from baseline (T1) to final follow-up (T4), with additional phase-specific improvements during prehabilitation (T1 → T2) and postoperative rehabilitation (T3 → T4). We further hypothesised that the pathway would be associated with favourable haemodynamic changes from T1 to T4, reflected by lower pre-6MWT resting and post-6MWT heart rate and reduced blood pressure. Finally, we hypothesised that patients would demonstrate substantial reductions in body mass, body mass index, and body circumferences (waist and chest) from T1 to T4.

## 2. Materials and Methods

### 2.1. KOS-BAR Programme Context

This study was conducted within the Polish Comprehensive Specialist Care in Bariatric Treatment programme (KOS-BAR). KOS-BAR operated in Poland between 2021 and 2025 as a nationwide bariatric care pathway financed by the National Health Fund (Narodowy Fundusz Zdrowia, NFZ). The programme covered eligibility assessment, surgery, and postoperative follow-up, and provided multidisciplinary care including preoperative diagnostics, perioperative support (prehabilitation, lifestyle and dietary education, psychological support), surgical treatment, and postoperative management, including rehabilitation and follow-up with a dietitian, physiotherapist, and psychologist [34]. The present retrospective analysis used anonymised programme records. The study protocol was approved by the university bioethics committee (KB/130/2024; 5 April 2024). Accordingly, physiotherapy should be interpreted as one component of the broader KOS-BAR pathway rather than as an isolated exposure.

### 2.2. Study Design and Setting

This was a single-centre retrospective medical-record review conducted at the Physiotherapy Unit of the University Clinical Centre in Gdańsk, Poland. The analysis was designed to describe within-patient changes observed during routine care and was not intended to estimate the independent causal effect of physiotherapy. Programme records were reviewed for the preoperative period and postoperative follow-up in accordance with programme guidance [34]. In routine practice, preoperative visits were conducted during surgical preparation, typically over 3–6 months between programme qualification and surgery. Postoperative visits were performed within up to 12 months after surgery as part of routine outcome monitoring.

### 2.3. Participants

The source population comprised adults enrolled in KOS-BAR and referred for bariatric surgery at the study centre. For this retrospective analysis, we reviewed records of patients qualified between January and November 2023 and selected a complete-case cohort of programme completers who had completed both supervised physiotherapy cycles and had paired T1 and T4 data available for the primary outcomes. The available retrospective dataset did not include a complete administrative registry of all patients initially eligible for KOS-BAR at the centre. Therefore, the total number and baseline characteristics of all eligible non-completers could not be determined.

Inclusion criteria were: (i) age 18–70 years; (ii) BMI > 30 kg/m^2^ at programme entry; and (iii) qualification for bariatric surgery according to routine KOS-BAR programme criteria. Inclusion additionally required completion of both 10-session physiotherapy cycles (preoperative and postoperative) and availability of paired T1 and T4 data for the primary outcomes. Exclusion criteria were BMI < 30 kg/m^2^, incomplete data for key outcomes required for analysis, or interruption of the 6MWT without a recorded walking distance.

**Figure 1:**
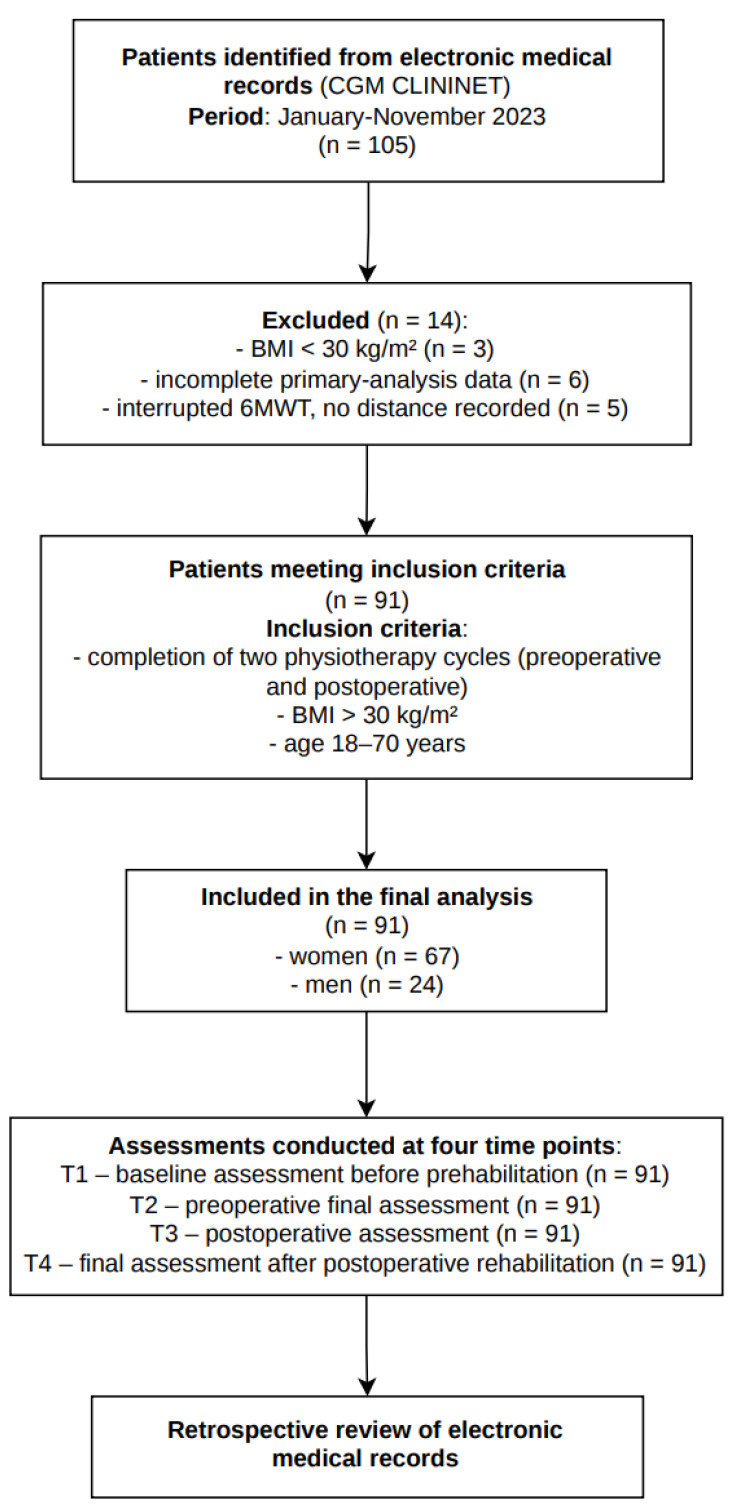
Flow diagram of available retrospective physiotherapy records, exclusions, intervention stages, and assessment points within the KOS-BAR programme. Note: The available retrospective dataset did not include all initially eligible KOS-BAR patients or structured data for non-completers; therefore, non-completer characteristics could not be compared.

Retrospective physiotherapy records were available for 105 patients (76 women and 29 men). Fourteen records were excluded: 3 patients had BMI < 30 kg/m^2^, 6 patients had incomplete data for the primary analyses, and 5 patients interrupted the 6 min walk test, with no walking distance recorded in the documentation. The final complete-case cohort included 91 patients (67 women and 24 men) who completed both physiotherapy cycles and had paired outcome data available (Figure 1).

This analysis therefore represents a selected complete-case cohort of programme completers with paired outcome data, and not the full population of all patients initially eligible for KOS-BAR at the centre. Because the study was based on routine clinical records, exposure to non-physiotherapy components of care, medication changes, and activity performed outside supervised sessions could not be fully quantified. In addition, because the excluded group was small and heterogeneous and several records lacked complete baseline or outcome data, formal comparison between included and excluded patients was not performed.

### 2.4. Timing of Assessments (T1–T4)

Patients underwent standardised physiotherapy evaluations at four time points: T1, before preoperative physiotherapy; T2, after preoperative physiotherapy; T3, after surgery and at the start of postoperative physiotherapy; and T4, after postoperative physiotherapy. Baseline assessments were performed directly before each physiotherapy cycle and follow-up assessments directly after each cycle. The preoperative physiotherapy stage was planned within a 3–6-month preoperative programme window and lasted approximately 3 months on average, although some patients required longer. The postoperative physiotherapy stage could be completed within the 12-month postoperative programme window and lasted approximately 5 months on average. These values should be interpreted as approximate programme-completion timing proxies derived from routine clinical scheduling rather than patient-level interval data. Exact individual dates required to calculate intervals between T1, T2, surgery, T3, and T4 were not consistently available in structured form in the retrospective physiotherapy dataset. Therefore, interval-specific median [IQR] values and covariate-adjusted analyses using time since surgery could not be performed.

### 2.5. Outcomes and Assessment Procedures

At each assessment, anthropometric outcomes included body mass, height, waist circumference, chest circumference, and calculated BMI. Functional capacity was assessed using the 6 min walk test (6MWT). Heart rate (HR) and peripheral oxygen saturation (SpO_2_) were recorded, and blood pressure (BP) was measured immediately before and immediately after the 6MWT using an automated oscillometric device (ELEKTRO-MED GERÄTE^®^, Medtronic, Meerbusch, Germany). Perceived exertion was rated immediately before and immediately after the 6MWT using the modified Borg CR10 scale (0–10). Based on a structured patient interview, additional information was recorded on leisure-time physical activity (yes/no), musculoskeletal complaints, and comorbidities. Leisure-time physical activity was self-reported and was not verified using objective monitoring or external records.

All assessments were performed in the same physiotherapy clinic using standardised procedures. Body mass was measured using the same calibrated scale across assessments. Waist and chest circumferences were measured using a non-elastic measuring tape. Waist circumference was measured at the level of the umbilicus with the participant standing upright, arms relaxed alongside the body, and feet together; the tape was placed horizontally and the measurement was taken at the end of a relaxed expiration, without intentional abdominal drawing-in or protrusion. Chest circumference was measured at the level of the axillary fossae using the same body position and the same phase of the respiratory cycle.

Heart rate, blood pressure, SpO_2_, and Borg score were recorded before and after the 6MWT as part of the standard clinical testing procedure. The pre-test heart-rate value was reported as pre-6MWT resting HR and should be interpreted as heart rate measured before the 6MWT, rather than as 24 h, morning, or wearable-derived resting heart rate.

The 6MWT was performed according to standardised procedures [35,36]. Before the test, resting HR, BP, and SpO_2_ were recorded, and participants rated perceived exertion on the 0–10 Borg scale. Immediately after completion, HR, BP, and SpO_2_ were re-measured, the total distance walked was recorded, and post-test perceived exertion was documented.

### 2.6. Physiotherapy Intervention

Patients completed two outpatient physiotherapy cycles: a preoperative prehabilitation cycle and a postoperative rehabilitation cycle. Each cycle comprised 10 supervised sessions delivered at the Physiotherapy Unit of the University Clinical Centre. All patients included in the analysed cohort completed both planned supervised cycles; therefore, supervised session completion was 10/10 sessions in the preoperative cycle and 10/10 sessions in the postoperative cycle for every included participant. Completion of both supervised cycles was an inclusion criterion; therefore, patients with early termination or suspension of the supervised programme were not included in the final complete-case cohort.

The programme was designed to allow up to three supervised sessions per week, but actual scheduling varied according to routine clinical availability and patient circumstances. Based on available programme records, the preoperative cycle lasted approximately 3 months on average, whereas the postoperative cycle lasted approximately 5 months on average.

Each supervised session lasted 60 min and included a 30 min group-based component and a 30 min individual component. The group-based component consisted of general conditioning exercises incorporating isometric and isotonic strengthening, breathing exercises, and circuit-style training. Common equipment included exercise sticks, balls, TheraBand^®^ resistance bands (Performance Health, Akron, OH, USA), and a multi-gym/weight station.

The individual aerobic component was performed using available rehabilitation equipment, including a treadmill, cycle ergometer, upper-limb ergometer, lower-limb rotor or semi-recumbent ergometer, and elliptical trainer. Exercise modality was selected individually according to patient tolerance, functional status, and concurrent musculoskeletal limitations. Equipment included ERGO-FIT devices (ERGOFIT GmbH, Pirmasens, Germany), an XRISE CYCLE cardiowise^®^ cycle ergometer (ERGOFIT GmbH, Pirmasens, Germany), and an ERM-200 device (ITAM, Zabrze, Poland).

According to the programme protocol, training intensity was prescribed individually for each patient at 60–80% of heart rate reserve (HRR) and 6–7 points on the 0–10 Borg scale. Any adverse events documented during supervised sessions were extracted from routine clinical records. However, because this was a retrospective medical-record study, session-by-session achieved intensity, duration spent on each aerobic modality, modality-specific progression, and physiological training responses were not available as structured quantitative variables.

Participants were also instructed to perform a home-exercise programme on non-clinic days [37,38]. Adherence to home-based exercise was assessed by verbal self-report to the physiotherapist during routine care. No objective monitoring or activity diaries were used; therefore, home-exercise frequency, duration, intensity, and adherence were not available as quantitative exposure data for analysis.

### 2.7. Other Programme Components

During the KOS-BAR programme, patients received multidisciplinary care delivered by a specialist team that included a surgeon, an internal medicine specialist and/or diabetologist, an anaesthesiologist, a medical rehabilitation specialist, a physiotherapist, a dietitian, a psychologist, a nurse, and a medical assistant, with additional specialist consultations as required. Alongside physiotherapy, patients underwent comprehensive preoperative preparation including clinical work-up and psychological support, and received structured education covering nutrition, physical activity, lifestyle modification, and the role of mental health in long-term weight management. Routine programme diagnostics included abdominal ultrasonography, polysomnography when indicated, gastroscopy, and laboratory testing including glucose and lipid profiles.

### 2.8. Statistical Analysis

All analyses were performed in Python (version 3.11) using pandas (version 2.2.0), NumPy (version 1.26.4), SciPy (version 1.12.0), and matplotlib (version 3.5.2). Statistical significance was set at α = 0.05. Normality of within-participant change scores was assessed using the Shapiro–Wilk test. Continuous outcomes are reported as mean ± standard deviation (SD) or median [interquartile range, IQR], as appropriate.

Because the study objective was to describe predefined within-patient changes across clinically relevant programme phases, analyses were based on paired within-participant comparisons rather than longitudinal mixed-effects modelling. Paired comparisons were conducted using the paired Student’s *t*-test when differences were approximately normally distributed and the Wilcoxon signed-rank test otherwise. For paired *t*-tests, effect size was quantified using Cohen’s dz.

For the primary confirmatory T1 vs. T4 comparisons, Bonferroni correction was applied for 15 outcomes (adjusted α = 0.05/15 = 0.0033). Multiplicity adjustment was restricted to the primary confirmatory analyses. All other analyses were considered exploratory and interpreted descriptively. Perioperative trajectory analyses across time points were descriptive; *p*-values for phase-specific paired comparisons (T2 vs. T1, T3 vs. T2, T4 vs. T3) are reported without multiplicity adjustment.

Exploratory associations between anthropometric change (weight loss and BMI reduction from T1 to T4) and changes in functional and physiological outcomes were assessed using Spearman’s rank correlation. Subgroup analyses by bariatric procedure were conducted for sleeve gastrectomy and mini-gastric bypass; SASI and Roux-en-Y gastric bypass were not analysed because of very small sample sizes (*n* = 2 and *n* = 1, respectively). Leisure-time physical activity was recorded as a binary variable (yes/no) at T1 and T4. Cardiological comorbidities were coded as a binary variable (present/absent) based on diagnoses documented in the medical record; because the underlying diagnoses were not uniformly detailed, this variable was analysed descriptively only. Because all included participants completed both supervised physiotherapy cycles and because quantitative data on home-exercise adherence, non-supervised physical activity, medication changes, dietary adherence, and non-completer characteristics were unavailable, adherence-stratified analyses, dose–response analyses, and covariate-adjusted sensitivity analyses for these factors were not performed. Similarly, because exact patient-level timing intervals between T1, T2, surgery, T3, and T4 were not consistently available in structured form, the time since surgery could not be incorporated as a covariate. Phase-specific comparisons, especially T2 vs. T3, should therefore be interpreted as descriptive within-patient changes rather than as adjusted estimates of surgery-, time-, or rehabilitation-specific effects.

## 3. Results

### 3.1. Participant Characteristics

A total of 91 patients were included: 67 women (73.6%) and 24 men (26.4%). Mean age was 43.3 ± 9.7 years (range 21–68). Median baseline BMI was 41.52 kg/m^2^ [38.40–45.25]. The most common bariatric procedure was sleeve gastrectomy (*n* = 76; 83.5%). Comorbidities documented in the medical records included mainly arterial hypertension (36/91, 39.6%), thyroid disorders including hypothyroidism or thyroidectomy (16/91, 17.6%), diabetes mellitus (14/91, 15.4%), insulin resistance (13/91, 14.3%), and asthma (10/91, 11.0%). Cardiological comorbidities were documented in 41 patients (45.1%), while 50 patients (54.9%) had no cardiological comorbidities documented. Because comorbidities were extracted from free-text medical-record entries rather than predefined diagnostic categories, these categories may overlap and are reported descriptively. As described above, these patients represent programme completers with paired outcome data; baseline characteristics of eligible non-completers were not available for comparison. Baseline sample characteristics are presented in Table 1.

### 3.2. Baseline Functional and Haemodynamic Status (T1)

At baseline (T1), exercise tolerance was assessed with the 6 min walk test (6MWT). The median distance was 510.0 m [481.0–540.0], with a mean of 504.7 ± 63.8 m (Table 2). Median pre-6MWT resting HR was 81.0 beats/min [72.0–91.0], and post-6MWT HR averaged 105.3 ± 21.8 beats/min. Resting blood pressure averaged 134.6 ± 16.0 mmHg (systolic) and 92.4 ± 11.5 mmHg (diastolic). Mean resting oxygen saturation was 98.2 ± 1.1%. Perceived exertion was low before the test (Borg 0.14 ± 0.50) and increased after the test (Borg 2.57 ± 0.94) (Table 2).

### 3.3. Changes from Baseline (T1) to Final Assessment (T4)

From T1 to T4, body mass, BMI, waist circumference, and chest circumference decreased (Table 3). Median body mass decreased from 120.0 [107.0–136.0] kg to 94.0 [84.5–103.0] kg (median Δ −26.0 kg [−32.0; −19.6], *p* < 0.001). Median BMI decreased from 41.52 [38.40–45.25] to 32.61 [30.02–35.64] kg/m^2^ (Δ −8.98 [−11.58; −6.99], *p* < 0.001). Waist circumference decreased by a median of 19.0 cm [−25.5; −15.0] and chest circumference by 13.0 cm [−16.0; −9.5] (both *p* < 0.001).

**Figure 2:**
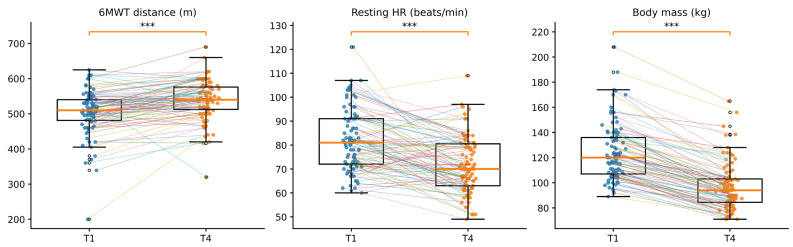
Changes in 6 min walk test (6MWT) distance, pre-6MWT resting heart rate (HR), and body mass from baseline (T1) to final follow-up (T4) within the multidisciplinary KOS-BAR pathway. Note: Blue and orange circles indicate individual values at T1 and T4, respectively; thin colored lines connect paired measurements from the same participant. Box-and-whisker plots display the median, interquartile range (IQR), and outliers. Significance was evaluated using the Bonferroni-adjusted threshold for Table 3 (α = 0.0033); *** *p* < 0.001.

Exercise tolerance (6MWT distance) increased between T1 and T4 (median Δ 30.0 m [10.0–60.0], *p* < 0.001) and remained statistically significant after Bonferroni correction (adjusted α = 0.0033; Figure 2, Table 3).

**Figure 3:**
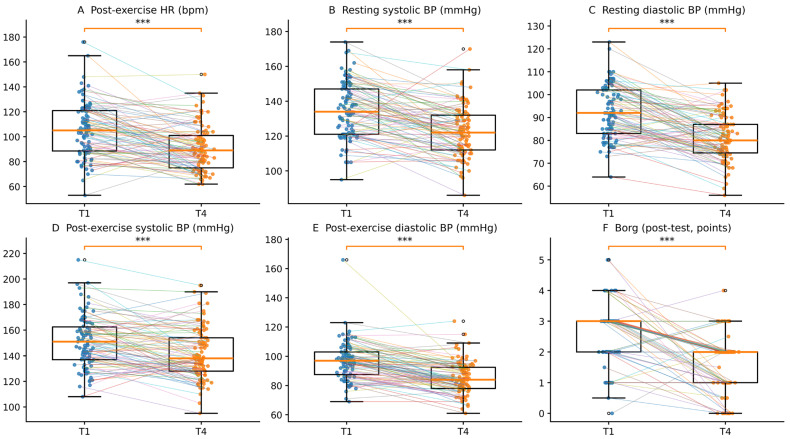
Changes in haemodynamic response to the 6 min walk test and perceived exertion from baseline (T1) to final follow-up (T4) within the multidisciplinary KOS-BAR pathway. Legend: Box-and-whisker plots show (**A**) post-exercise heart rate, (**B**) resting systolic blood pressure, (**C**) resting diastolic blood pressure, (**D**) post-exercise systolic blood pressure, (**E**) post-exercise diastolic blood pressure, and (**F**) Borg rating of perceived exertion after the 6 min walk test. Boxes indicate the interquartile range (IQR) with the median; whiskers represent 1.5 × IQR and points indicate outliers. Thin lines connect paired observations (T1 to T4). Significance was evaluated using the Bonferroni-adjusted threshold for Table 3 (α = 0.0033); *** *p* < 0.001. Abbreviations: BP, blood pressure; bpm, beats per minute; IQR, interquartile range.

Haemodynamic parameters improved (Figure 3, Table 3). Pre-6MWT resting HR decreased (median Δ −9.0 bpm [−18.5; 1.5], *p* < 0.001). Post-6MWT HR also decreased (mean Δ −14.7 ± 21.6 bpm, *p* < 0.001; Cohen’s dz = −0.680). Resting systolic BP decreased (Δ −11.8 ± 16.5 mmHg, *p* < 0.001; dz = −0.712) and resting diastolic BP decreased (Δ −11.2 ± 12.3 mmHg, *p* < 0.001; dz = −0.915). Post-6MWT systolic BP decreased (Δ −9.3 ± 18.7 mmHg, *p* < 0.001; dz = −0.499), and post-6MWT diastolic BP decreased (median Δ −12.0 mmHg [−18.0; −4.0], *p* < 0.001).

SpO_2_ changed minimally; nominal differences were observed for resting (*p* = 0.006) and post-6MWT SpO_2_ (*p* = 0.013), but these did not remain significant after Bonferroni correction. Borg score before the 6MWT did not change (*p* = 0.086), whereas the Borg score after the 6MWT decreased from 3.0 [2.0–3.0] to 2.0 [1.0–2.0] (median Δ −1.0 point [−1.5; 0.0], *p* < 0.001), indicating lower perceived exertion at follow-up.

**Table 3:** Changes in anthropometric, functional, and haemodynamic outcomes (T1 vs. T4).

Parameter	T1	T4	Change (Δ)	95% CI for Δ	Test	*p*-Value	Effect Size *
	Anthropometric parameters						
Body mass (kg)	120.0 [107.0–136.0]	94.0 [84.5–103.0]	−26.0 [−32.0 to −19.6]	−28.0 to −23.0	Wilcoxon	<0.001	—
BMI (kg/m^2^)	41.52 [38.40–45.25]	32.61 [30.02–35.64]	−8.98 [−11.58 to −6.99]	−9.80 to −8.11	Wilcoxon	<0.001	—
Waist circumference (cm)	121.0 [112.5–131.5]	102.0 [94.0–109.5]	−19.0 [−25.5 to −15.0]	−21.0 to −18.0	Wilcoxon	<0.001	—
Chest circumference (cm)	116.0 [109.0–125.0]	102.0 [99.0–109.5]	−13.0 [−16.0 to −9.5]	−14.0 to −12.0	Wilcoxon	<0.001	—
6MWT distance (m)	510.0 [481.0–540.0]	540.0 [512.5–576.0]	30.0 [10.0 to 60.0]	20.0 to 40.0	Wilcoxon	<0.001	—
	Haemodynamic parameters						
Pre-6MWT resting HR (beats/min)	81.0 [72.0–91.0]	70.0 [63.0–80.5]	−9.0 [−18.5 to 1.5]	−12.0 to −6.0	Wilcoxon	<0.001	—
Post-6MWT HR (beats/min)	105.3 ± 21.8	90.6 ± 18.5	−14.7 ± 21.6	−19.1 to −10.2	Paired *t*-test	<0.001	−0.680
Resting systolic BP (mmHg)	134.6 ± 16.0	122.9 ± 14.7	−11.8 ± 16.5	−15.2 to −8.3	Paired *t*-test	<0.001	−0.712
Resting diastolic BP (mmHg)	92.4 ± 11.5	81.1 ± 10.0	−11.2 ± 12.3	−13.8 to −8.7	Paired *t*-test	<0.001	−0.915
Post-6MWT systolic BP (mmHg)	151.1 ± 20.2	141.7 ± 20.0	−9.3 ± 18.7	−13.2 to −5.4	Paired *t*-test	<0.001	−0.499
Post-6MWT diastolic BP (mmHg)	97.0 [87.5–103.0]	84.0 [78.0–92.5]	−12.0 [−18.0 to −4.0]	−14.0 to −9.0	Wilcoxon	<0.001	—
Resting SpO_2_ (%)	98.0 [98.0–99.0]	99.0 [98.0–99.0]	0.0 [0.0 to 1.0]	0.0 to 1.0	Wilcoxon	0.006	—
Post-6MWT SpO_2_ (%)	99.0 [98.0–99.0]	99.0 [98.0–99.0]	0.0 [0.0 to 1.0]	0.0 to 0.0	Wilcoxon	0.013	—
Pre-6MWT Borg score	0.0 [0.0–0.0]	0.0 [0.0–0.0]	0.0 [0.0 to 0.0]	0.0 to 0.0	Wilcoxon	0.086	—
Post-6MWT Borg score	3.0 [2.0–3.0]	2.0 [1.0–2.0]	−1.0 [−1.5 to 0.0]	−1.0 to −0.5	Wilcoxon	<0.001	—

Note: Data are presented as mean ± SD or median [IQR], depending on distribution and statistical test. Δ = T4 − T1. The 95% CI for Δ denotes the *t*-based 95% CI for the mean paired change for paired *t*-tests and the percentile bootstrap 95% CI for the median paired change for Wilcoxon comparisons. * Effect size: Cohen’s dz for paired *t*-tests. Bonferroni-adjusted significance threshold for Table 3 comparisons: α = 0.05/15 = 0.0033.

### 3.4. Changes Across Perioperative Time Points (T1–T4)

**Figure 4:**
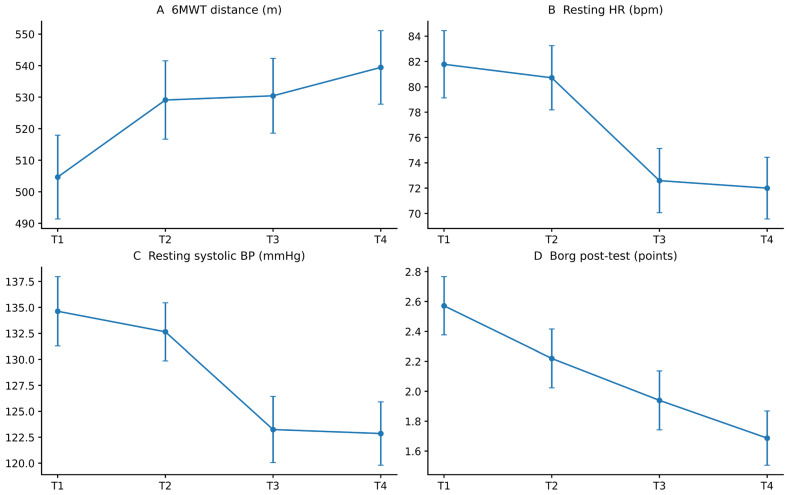
Perioperative trajectories of exercise tolerance and cardiometabolic responses across four time points within the multidisciplinary KOS-BAR pathway. Legend: Panels show mean values with 95% confidence intervals for (**A**) 6 min walk test (6MWT) distance, (**B**) resting heart rate (HR), (**C**) resting systolic blood pressure (BP), and (**D**) Borg rating of perceived exertion after the 6MWT. Time points represent prehabilitation baseline (T1), end of prehabilitation (T2), postoperative rehabilitation baseline (T3), and end of postoperative rehabilitation (T4). Abbreviations: 6MWT, 6 min walk test; HR, heart rate; BP, blood pressure.

Across the four time points, exercise tolerance assessed by the 6 min walk test (6MWT) improved (Figure 4). The 6MWT distance increased during prehabilitation (T1 → T2: 504.7 ± 63.8 m to 529.1 ± 59.7 m; *p* < 0.001), showed no change across the perioperative transition (T2 → T3: *p* = 0.687), and increased further during postoperative rehabilitation (T3 → T4: 530.4 ± 56.9 m to 539.4 ± 56.0 m; *p* < 0.001) (Table 4). Resting diastolic blood pressure decreased during prehabilitation (T1 → T2: 92.4 ± 11.5 to 88.4 ± 9.5 mmHg; *p* < 0.001), whereas resting heart rate and resting systolic blood pressure did not change significantly within this cycle (*p* > 0.05). In contrast, marked reductions in resting heart rate, post-6MWT heart rate, and both systolic and diastolic blood pressure were observed after surgery (T2 → T3: *p* < 0.001; Table 4). Post-exercise perceived exertion decreased during prehabilitation (T1 → T2: 2.57 ± 0.94 to 2.22 ± 0.94; *p* = 0.002) and decreased further during postoperative rehabilitation (T3 → T4: 1.94 ± 0.95 to 1.69 ± 0.87; *p* < 0.001). Changes in resting SpO_2_ and pre-6MWT Borg score were small and not clinically meaningful; nominal *p*-values were observed (e.g., resting SpO_2_ T1 → T2 *p* = 0.049) (Table 4).

**Table 4:** Functional and haemodynamic parameters across four time points (T1–T4).

Parameter	T1 (Prehabilitation Baseline)	T2 (Prehabilitation End)	T3 (Postoperative Rehabilitation Baseline)	T4 (Postoperative Rehabilitation End)	*p* (T2 vs. T1)	*p* (T3 vs. T2)	*p* (T4 vs. T3)
6MWT distance (m)	504.7 ± 63.8	529.1 ± 59.7	530.4 ± 56.9	539.4 ± 56.0	<0.001	0.687	<0.001
Pre-6MWT resting HR (beats/min)	81.8 ± 12.8	80.7 ± 12.2	72.6 ± 12.2	72.0 ± 11.7	0.381	<0.001	0.528
Post-6MWT HR (beats/min)	105.3 ± 21.8	103.7 ± 16.5	95.0 ± 19.4	90.6 ± 18.5	0.286	<0.001	0.002
Resting systolic BP (mmHg)	134.6 ± 16.0	132.6 ± 13.4	123.2 ± 15.3	122.9 ± 14.7	0.151	<0.001	0.186
Resting diastolic BP (mmHg)	92.4 ± 11.5	88.4 ± 9.5	81.7 ± 10.2	81.1 ± 10.0	<0.001	<0.001	0.189
Resting SpO_2_ (%)	98.19 ± 1.11	98.43 ± 0.87	98.43 ± 0.99	98.56 ± 0.88	0.049	0.795	0.290
Pre-6MWT Borg score	0.14 ± 0.50	0.08 ± 0.29	0.12 ± 0.49	0.05 ± 0.35	0.413	0.596	0.112
Post-6MWT Borg score	2.57 ± 0.94	2.22 ± 0.94	1.94 ± 0.95	1.69 ± 0.87	0.002	0.030	<0.001

Note: Values are mean ± SD. *p*-values refer to paired within-participant comparisons within each phase (prehabilitation: T2 vs. T1; perioperative transition: T3 vs. T2; postoperative rehabilitation: T4 vs. T3). These phase-specific analyses were exploratory and were not adjusted for multiplicity. Abbreviations: 6MWT, 6 min walk test; HR, heart rate; BP, blood pressure; SpO_2_, peripheral oxygen saturation.

### 3.5. Exploratory Correlation Analysis

Greater weight loss from T1 to T4 was weakly associated with larger improvement in 6MWT distance (Spearman r = 0.251, *p* = 0.016). BMI reduction showed a similar weak association with 6MWT improvement (r = 0.226, *p* = 0.031). No significant associations were observed between weight loss and changes in resting heart rate, post-6MWT heart rate, resting systolic blood pressure, resting SpO_2_, or post-test Borg score (all *p* > 0.05) (Table 5). After Bonferroni correction for seven correlation tests (α = 0.007), these associations did not remain statistically significant.

**Table 5:** Exploratory correlations between anthropometric change and functional or physiological change (T1–T4).

Independent Variable	Dependent Variable	Spearman r	*p*-Value
Weight loss (kg)	Change in 6MWT distance (m)	0.251	0.016
BMI reduction (kg/m^2^)	Change in 6MWT distance (m)	0.226	0.031
Weight loss (kg)	Change in pre-6MWT resting HR (beats/min)	−0.111	0.297
Weight loss (kg)	Change in post-6MWT HR (beats/min)	0.006	0.952
Weight loss (kg)	Change in resting systolic BP (mmHg)	−0.106	0.318
Weight loss (kg)	Change in resting SpO_2_ (%)	0.054	0.611
Weight loss (kg)	Change in post-6MWT Borg score	−0.079	0.455

Note: Weight loss and BMI reduction were defined as positive values (T1 − T4). Dependent variables were defined as change from baseline (T4 − T1); therefore, positive values indicate increases over time (e.g., higher 6MWT distance), whereas negative values indicate decreases (e.g., lower heart rate or Borg score). Abbreviations: BMI, body mass index; 6MWT, 6 min walk test; HR, heart rate; BP, blood pressure; SpO_2_, peripheral oxygen saturation. These exploratory correlations did not remain statistically significant after Bonferroni correction.

### 3.6. Self-Reported Physical Activity and Comorbidities

Self-reported leisure-time physical activity (binary yes/no) remained low, increasing from baseline to follow-up: 4.4% (4/91) at baseline versus 11.0% (10/91) at follow-up. Accordingly, 95.6% (87/91) reported no leisure-time physical activity at baseline compared with 89.0% (81/91) at follow-up. Most participants remained inactive (78/91, 85.7%), while nine participants reported initiating leisure-time activity (no → yes) and three reported stopping (yes → no). Cardiological comorbidities were recorded in 41 patients (45.1%), while 50 patients (54.9%) had no cardiological comorbidities recorded. Cardiological comorbidities were extracted from the dedicated binary field in the medical record; because the specific conditions were not predefined or consistently detailed, these data are presented descriptively.

### 3.7. Subgroup Analyses by Procedure Type

In procedure subgroup analyses, both sleeve gastrectomy (*n* = 76) and mini-gastric bypass (*n* = 12) groups showed significant reductions in body mass and BMI between T1 and T4 (all *p* < 0.001). Both subgroups also demonstrated improvements in 6MWT distance from T1 to T4 (sleeve: 509.1 ± 53.7 m to 538.9 ± 50.1 m; *p* < 0.001; mini-gastric bypass: 466.5 ± 104.9 m to 532.6 ± 80.1 m; *p* = 0.012) (Table 6). Due to very small sample sizes, SASI (*n* = 2) and Roux-en-Y gastric bypass (*n* = 1) subgroups were not analysed. Subgroup analyses were hypothesis-generating only.

## 4. Discussion

The literature includes studies evaluating prehabilitation before bariatric surgery [24,26,32] and, separately, postoperative rehabilitation [31,39,40]. Most published work treats these stages as distinct interventions and rarely evaluates perioperative physiotherapy as a continuous care pathway spanning prehabilitation and postoperative rehabilitation [39,40]. In Poland, physiotherapy within bariatric care remains under-represented in the scientific literature. To date, one Polish study has assessed supervised preoperative exercise and its effects on selected functional outcomes [24]. To our knowledge, no published Polish studies have examined a continuous perioperative multidisciplinary KOS-BAR pathway with embedded physiotherapy delivered before and after bariatric surgery within the KOS-BAR model. The present analysis therefore adds real-world evidence on outcomes observed across a multidisciplinary bariatric pathway in which physiotherapy was embedded before and after surgery. Because physiotherapy exposure was not isolated from surgery, dietary counselling, psychological support, medical management, and postoperative recovery, the observed changes cannot be attributed to physiotherapy independently.

### 4.1. Alignment with Aims and Hypotheses

The aim of this study was to evaluate outcomes observed in patients completing preoperative and postoperative physiotherapy embedded within the multidisciplinary KOS-BAR bariatric pathway. Overall, completion of the pathway was accompanied by improvements in anthropometric status, haemodynamic parameters, and exercise tolerance across follow-up.

These hypothesis-level findings should be interpreted as within-patient changes over time in a multidisciplinary surgical pathway, not as evidence that physiotherapy independently caused the observed anthropometric or haemodynamic changes. The hypothesis of favourable haemodynamic changes was also supported, as resting and post-exercise heart rate and blood pressure were lower at follow-up; however, phase-specific analyses suggest that the largest haemodynamic improvements occurred after surgery (T2 → T3) rather than during prehabilitation alone. Exercise tolerance improved over time: 6MWT distance increased during prehabilitation (T1 → T2), remained stable across the perioperative transition (T2 → T3), and improved further during postoperative rehabilitation (T3 → T4). These findings should be interpreted as associations observed within a multidisciplinary programme that includes surgery and other concurrent care components.

### 4.2. The Role of Prehabilitation

Functional assessment is a key component of preparation for bariatric surgery because it enables objective quantification of exercise tolerance, monitoring of change over time, and identification of domains that may require optimisation prior to surgery [20,41]. In this study, use of a consistent assessment protocol at four time points (T1–T4) enabled separate description of changes occurring during prehabilitation (T1 → T2), the perioperative transition (T2 → T3), and postoperative rehabilitation (T3 → T4).

During prehabilitation, exercise tolerance improved, as reflected by a higher 6MWT distance, and perceived exertion after the test decreased, indicating lower symptom burden for a comparable functional task. This pattern is consistent with evidence suggesting that structured preoperative physiotherapy may enhance functional capacity before bariatric surgery [26]. However, because this was an uncontrolled retrospective analysis and because home-exercise adherence and non-supervised activity were not objectively monitored, these preoperative changes should be interpreted as associations observed during the prehabilitation phase rather than as definitive training effects. In contrast, haemodynamic changes during prehabilitation were more limited: resting diastolic blood pressure decreased, whereas resting heart rate and resting systolic blood pressure did not change significantly within this phase. This suggests that prehabilitation in this pathway was primarily associated with improved functional performance and perceived exertion, with selective haemodynamic improvement that may still be potentially clinically relevant for surgical preparation [24,42,43]. Given the observational design, these data do not allow causal conclusions regarding mechanisms or the contribution of individual programme components.

### 4.3. Dynamics of Change During Prehabilitation and Postoperative Rehabilitation

Analysis across four time points provides insight into when key changes emerged and whether they were maintained. Prehabilitation was associated with early functional improvements, with increased 6MWT distance and reduced post-test perceived exertion [24,26]. The stability of 6MWT distance between the end of prehabilitation and the start of postoperative rehabilitation (T2 → T3) may indicate maintenance of functional capacity across the perioperative transition despite the physiological stress of surgery and early recovery [24,26]. In contrast, marked reductions in heart rate and blood pressure were observed after surgery (T2 → T3), consistent with reduced cardiovascular load accompanying substantial weight loss and postoperative recovery. However, these changes may also have been influenced by medication adjustments, improved management of obesity-related comorbidities, dietary adherence, and broader clinical follow-up, none of which were available as structured quantitative variables in the retrospective dataset. Finally, further gains in 6MWT distance together with lower perceived exertion during postoperative rehabilitation (T3 → T4) are consistent with continued functional improvement within the programme context [29,40].

### 4.4. Anthropometric Changes

Substantial reductions in body mass, BMI, waist circumference, and chest circumference were observed across the KOS-BAR pathway. These changes are most plausibly explained primarily by bariatric surgery and the broader multidisciplinary weight-loss programme, including dietary and medical management, rather than by physiotherapy alone. Median body mass decreased from 120.0 kg to 94.0 kg (≈22% relative reduction). This pattern is consistent with the established effectiveness of bariatric surgery in producing large weight loss and reducing central adiposity, and it aligns with prior reports describing favourable functional and physiological profiles in patients undergoing bariatric treatment within structured care pathways [24,33]. Reductions in body mass and circumferences may translate into lower mechanical loading of the lower-limb joints and spine, facilitating mobility and supporting functional independence [14,15,16].

### 4.5. Haemodynamic Changes and the Role of Weight Loss

Reductions in heart rate and blood pressure observed both at rest and after the 6MWT indicate a favourable shift in cardiovascular status across the perioperative period. Such changes are compatible with improved cardiorespiratory efficiency and cardiovascular adaptation [44,45]. Decreases in systolic and diastolic blood pressure may reflect reduced cardiovascular load and improved haemodynamic regulation [45,46]. The direction of these effects is consistent with reports from prehabilitation studies in bariatric populations showing that structured preoperative exercise can improve cardiorespiratory outcomes [24,26,42,43].

In exploratory analyses, weight loss was not significantly associated with changes in haemodynamic outcomes. This does not support a simple dose–response relationship between the magnitude of weight reduction and cardiovascular change in this cohort and suggests that factors beyond weight loss alone (e.g., participation in structured exercise and broader perioperative care) may have contributed. Similar observations have been reported in studies indicating that physical activity improves cardiorespiratory fitness independent of weight loss magnitude [47,48]. Given the retrospective design, lack of objective physical-activity quantification, and potential confounding from concurrent surgical and medical management (including medication changes), these findings should be interpreted as associations rather than evidence of causal mechanisms.

### 4.6. Exercise Tolerance

The observed improvement in 6MWT distance (median +30 m from T1 to T4) should be interpreted cautiously. The 6MWT distance is a pragmatic, validated measure of submaximal functional capacity and has been widely used in patients with severe obesity and bariatric surgery candidates. The mean distance in the present cohort increased from 504.7 ± 63.8 m at T1 to 539.4 ± 56.0 m at T4. For example, de Souza et al. [49] reported lower 6MWT distances in bariatric surgery patients, increasing from 381.9 ± 49.3 m preoperatively to 467.8 ± 40.3 m postoperatively, whereas Maniscalco et al. reported an increase from 475.7 m before laparoscopic adjustable gastric banding to 626.3 m one year after surgery [50]. Compared with these reports, the present cohort had relatively preserved baseline walking capacity, which may partly explain the smaller absolute gain and possible ceiling effect. A bariatric-surgery-specific MCID for the 6MWT is not firmly established. In a systematic review of adults with pathology, Bohannon and Crouch [51] reported that anchor-based MCID estimates with acceptable ROC performance ranged from 14.0 to 30.5 m and cautiously suggested that changes exceeding 30.5 m may be considered clinically meaningful. Therefore, the median improvement observed in the present cohort is close to the upper boundary of published MCID estimates from other clinical populations, but this interpretation remains indirect and should not be treated as confirmation of a bariatric-specific MCID. This gain occurred alongside reduced post-test perceived exertion, supporting an overall improvement in exercise tolerance. The findings align with evidence that supervised exercise interventions in bariatric candidates can improve functional capacity preoperatively, with Polish data indicating that structured physiotherapy within prehabilitation improves functional status before surgery [24,26,30,51]. They are also consistent with reports that postoperative rehabilitation is associated with improved physical function and quality of life compared with patients who do not engage in structured rehabilitation, although causal attribution remains limited in real-world observational designs [33]. The improvement also coincided with substantial weight loss and lower post-test Borg scores, suggesting that reduced mechanical and perceptual burden may have contributed alongside any training-related adaptation. A repeated-test or learning effect cannot be excluded.

### 4.7. Study Limitations

Interpretation must be tempered by several limitations. First, the retrospective design and lack of a contemporaneous control group preclude causal attribution of observed changes to physiotherapy. Improvements may reflect the combined effects of surgery, usual multidisciplinary care, medication adjustments, regression to the mean, and time. In addition, the study was based on available retrospective physiotherapy records rather than a complete administrative registry of all patients initially enrolled in KOS-BAR at the centre. Therefore, the full denominator of eligible patients and the characteristics of all non-completers could not be determined. Although exclusion reasons were identified for the 14 records excluded from the available dataset (BMI < 30 kg/m^2^, incomplete primary-analysis data, or interrupted 6MWT without recorded distance), formal comparison between included and excluded patients was not performed because the excluded group was small, heterogeneous, and incompletely documented. This limits assessment of selection and completion bias, and the results may overestimate achievable outcomes in the broader bariatric population.

Second, the study relied on routinely collected clinical data, which limited control over potential sources of bias and increased the risk of unmeasured confounding. Important potential confounders, including medication changes, dietary adherence, diabetes and hypertension management, psychological support, and broader lifestyle modification, were not available as structured quantitative variables. These factors may have contributed particularly to anthropometric and haemodynamic changes and may have biased the observed associations away from the null.

Third, although all included patients completed the planned supervised sessions, detailed exercise-dose data were unavailable. Exact weekly session frequency, session-by-session achieved intensity, duration spent on each aerobic modality, modality-specific progression, non-supervised physical activity, and home-exercise adherence were not objectively recorded as structured quantitative variables. Home-exercise adherence was assessed only by verbal self-report during routine care. Leisure-time activity was captured by self-report only, typically without consistent detail on frequency, duration, or intensity, and therefore could not be incorporated as a quantitative exposure; analyses were restricted to a binary indicator (yes/no). Consequently, dose–response relationships and adherence-stratified analyses could not be performed. VO_2_max and post-exercise heart-rate recovery were not assessed because cardiopulmonary exercise testing, gas-exchange analysis, standardised workload protocols, and standardised post-exercise heart-rate recovery measurements were not part of routine physiotherapy documentation. Although ergometers were used as training modalities, they were not used within a standardised exercise-testing protocol that would allow valid retrospective estimation of VO_2_max.

Fourth, programme-completion timing was available only as an approximate clinical scheduling proxy. The preoperative stage lasted approximately 3 months on average, whereas the postoperative stage lasted approximately 5 months on average within the 12-month postoperative programme window. Exact patient-level intervals between T1, T2, surgery, T3, and T4 were not consistently available in structured form. Consequently, interval-specific median [IQR] values could not be reported and time since surgery could not be included as a covariate. This limits interpretation of phase-specific changes, particularly the haemodynamic changes observed between T2 and T3, which may reflect surgery, postoperative recovery, weight loss, medication changes, elapsed time, or broader clinical management rather than the rehabilitation protocol itself.

Finally, although perioperative trajectories were described using repeated assessments (T1–T4), follow-up was limited to the programme window and durability of effects beyond programme completion cannot be inferred. Prospective studies—ideally controlled, with objective activity monitoring, structured medication and dietary data, patient-level timing intervals, and longer follow-up—are needed to quantify the independent contribution of perioperative physiotherapy, identify which programme components drive change, and evaluate maintenance of functional gains after discharge. Nonetheless, these real-world data may inform optimisation of bariatric care pathways and support development of more explicit physiotherapy standards within Polish bariatric services and comparable programmes elsewhere.

### 4.8. Practical Implications

From a clinical perspective, these findings support the use of repeated functional monitoring and structured physiotherapy within multidisciplinary bariatric care, rather than supporting physiotherapy as an isolated causal intervention. In this cohort, prehabilitation was most clearly associated with improved walking capacity and lower perceived exertion before surgery, whereas the largest haemodynamic changes were observed after surgery within the limits of the uncontrolled retrospective design. Postoperative rehabilitation may then help consolidate and extend functional gains. In practice, progression may be guided more appropriately by repeated objective assessment than by time alone. Future prospective studies should consider direct cardiopulmonary exercise testing or VO_2_ estimation methods validated specifically for bariatric surgery populations.

## 5. Conclusions

In this retrospective complete-case cohort, patients completing the multidisciplinary KOS-BAR pathway with embedded perioperative physiotherapy showed favourable anthropometric, functional, perceived-exertion, and haemodynamic changes. The findings support systematic functional monitoring and structured physiotherapy as practical components of multidisciplinary bariatric care. Phase-specific analyses suggested that walking capacity improved mainly during prehabilitation, whereas the largest haemodynamic changes occurred after surgery. Because the study was uncontrolled, retrospective, and restricted to programme completers, these changes cannot be attributed to physiotherapy alone. Prospective controlled studies with objective activity monitoring, detailed timing data, and cardiorespiratory fitness assessment are needed to determine which pathway components drive recovery.

## Figures and Tables

**Table 1:** Characteristics of the study sample.

Characteristic	Value
Demographics	
Age (years), mean ± SD	43.3 ± 9.7
Age range (years)	21–68
Sex, *n* (%)	
Women	67 (73.6)
Men	24 (26.4)
Baseline parameters	
Body mass (kg), median [IQR]	120.0 [107.0–136.0]
BMI (kg/m^2^), median [IQR]	41.52 [38.40–45.25]
Waist circumference (cm), median [IQR]	121.0 [112.5–131.5]
Chest circumference (cm), median [IQR]	116.0 [109.0–125.0]
Bariatric procedure, *n* (%)	
Sleeve gastrectomy	76 (83.5)
Mini-gastric bypass	12 (13.2)
SASI	2 (2.2)
Roux-en-Y gastric bypass	1 (1.1)

Note: Continuous variables are presented as mean ± SD or median [IQR] depending on distribution. Categorical variables are presented as *n* (%). Abbreviations: BMI, body mass index; IQR, interquartile range; SD, standard deviation; SASI, single-anastomosis sleeve ileal bypass.

**Table 2:** Baseline exercise tolerance and haemodynamic parameters (T1).

Parameter	Value
Exercise tolerance	
6MWT distance (m), median [IQR]	510.0 [481.0–540.0]
6MWT distance (m), mean ± SD	504.7 ± 63.8
Heart rate (HR)	
Pre-6MWT resting HR (beats/min), median [IQR]	81.0 [72.0–91.0]
Post-6MWT HR (beats/min), mean ± SD	105.3 ± 21.8
Blood pressure (BP)	
Resting systolic BP (mmHg), mean ± SD	134.6 ± 16.0
Resting diastolic BP (mmHg), mean ± SD	92.4 ± 11.5
Oxygen saturation (SpO_2_)	
Resting SpO_2_ (%), mean ± SD	98.2 ± 1.1
Perceived exertion (Borg scale)	
Pre-6MWT Borg score, mean ± SD	0.14 ± 0.50
Post-6MWT Borg score, mean ± SD	2.57 ± 0.94

Note: Abbreviations: 6MWT, 6 min walk test; HR, heart rate; BP, blood pressure; SpO_2_, peripheral oxygen saturation; SD, standard deviation; IQR, interquartile range.

**Table 6:** Exploratory outcomes by bariatric procedure type (T1 vs. T4).

Parameter	Sleeve Gastrectomy (*n* = 76) T1	Sleeve Gastrectomy (*n* = 76) T4	*p*-Value (T4 vs. T1) Sleeve	Mini-Gastric Bypass (*n* = 12) T1	Mini-Gastric Bypass (*n* = 12) T4	*p*-Value (T4 vs. T1) MGB
Body mass (kg), mean ± SD	122.5 ± 22.8	95.2 ± 18.4	<0.001	132.3 ± 21.8	106.0 ± 19.6	<0.001
BMI (kg/m^2^), mean ± SD	42.4 ± 5.9	33.0 ± 5.4	<0.001	43.4 ± 6.1	34.7 ± 5.4	<0.001
6MWT distance (m), mean ± SD	509.1 ± 53.7	538.9 ± 50.1	<0.001	466.5 ± 104.9	532.6 ± 80.1	0.012
Body mass loss (kg), mean ± SD (T1 − T4)	27.4 ± 11.7	—	—	26.4 ± 7.1	—	—
BMI reduction (kg/m^2^), mean ± SD (T1 − T4)	9.38 ± 3.58	—	—	8.68 ± 2.42	—	—
6MWT change (m), mean ± SD (T4 − T1)	29.8 ± 50.3	—	—	66.1 ± 76.6	—	—

Note: Values are mean ± SD. SASI (*n* = 2) and Roux-en-Y gastric bypass (*n* = 1) were not analysed due to insufficient sample size. Subgroup analyses were exploratory; *p*-values are reported without multiplicity adjustment.

## Data Availability

The data presented in this study are not publicly available due to ethical and privacy restrictions, as they were derived from anonymised routine clinical records from the KOS-BAR programme at the University Clinical Centre in Gdańsk. De-identified data may be made available from the corresponding author upon reasonable request and subject to approval by the relevant institutional authorities and ethics requirements.
